# Comparison between national and PAHO criteria for assessing the nutritional profile of foods intended for children aged 0–3 years marketed in Lima, Peru

**DOI:** 10.3389/fnut.2025.1592172

**Published:** 2025-09-09

**Authors:** Adolfo Aramburu, Jackeline Lizet Chupica-Leon, Beatriz Quispe-Quille, Julie Mariaca, Patricia Velarde-Delgado, Gilmer Solis-Sanchez, Bladimir Morales-Cahuancama, Fernando Bravo-Rebatta, María Elena Ugaz, Fredy Polo-Campos

**Affiliations:** ^1^Directorate of Health Promotion, Ministry of Health, Lima, Peru; ^2^Faculty of Science Health, Peruvian University of Applied Sciences, Lima, Peru; ^3^United Nations Children's Fund (UNICEF), Lima, Peru; ^4^National Center for Food, Nutrition and Healthy Living, National Institute of Health, Lima, Peru

**Keywords:** ultra-processed foods, infant food, breastmilk substitutes, infant formula, food labeling, legislation food, supermarkets

## Abstract

**Introduction:**

The rising consumption of industrialized infant foods and breastmilk substitutes during early childhood has raised concerns due to their suboptimal nutritional composition, which may contribute to the development of risk factors for non-communicable diseases. This study aimed to assess and compare the nutritional profile of foods intended for children aged 0–3 years marketed in Lima, Peru, using national criteria and the nutrient profile model developed by the Pan American Health Organization (PAHO).

**Methods:**

This cross-sectional, descriptive, and observational study analyzed products purchased from selected supermarkets and pharmacies in metropolitan Lima between September and October 2023. Nutritional labeling information was examined, and the nutritional profile was assessed using the nutrient profile models of Peru (Law No. 30021) and PAHO.

**Results:**

A total of 64 products were analyzed, including 39 breastmilk substitutes, 13 infant cereals, and 12 pureed foods. According to the parameters established by Law No. 30021, 74.5, 1.6, and 37.3% of products were high in total sugars, sodium, and saturated fats, respectively. According to PAHO parameters, the proportion of products classified as high in added sugars, sodium, and saturated fats increased to 83, 6.3, and 41.3%, respectively. Breastmilk substitutes were the main contributors to excessive saturated fat and total sugar content. Additionally, the presence of added sugars was identified in the ingredient lists of 84% of the products, corresponding to 100% of breastmilk substitutes, 53.9% of infant cereals, and 66.7% of pureed foods.

**Conclusion:**

The findings indicate that most breastmilk substitutes and foods marketed for children under three years old in metropolitan Lima have an unhealthy nutritional profile. Furthermore, the use of the technical parameters proposed by PAHO allowed for the identification of a higher number of products high in critical nutrients compared to the criteria established by Law No. 30021.

## 1 Introduction

Non-communicable diseases (NCDs) are the leading cause of death and disability globally, particularly affecting low- and middle-income countries (1, 2). Many of these diseases originate in early childhood, a stage during which inadequate diets can lead to childhood obesity, a critical determinant associated with a higher risk of developing NCDs throughout life, including cardiovascular diseases, type 2 diabetes, and certain cancers (3, 4).

Likewise, early childhood is a critical window for the development of taste preferences and the establishment of dietary patterns that often track into adulthood (5, 6). This formative period of flavor learning is highly susceptible to the surrounding food environment. It is precisely during this stage that the increasing availability and consumption of nutritionally suboptimal, ultra-processed foods (UPFs) can shape the palate toward unhealthy profiles (7). Therefore, ensuring the provision of diverse, safe, and nutritious foods is essential not only for meeting immediate nutritional requirements but also for actively fostering healthy eating patterns from the beginning of life (8, 9).

However, traditional infant feeding patterns centered on minimally processed foods are undergoing a continuous transition toward a higher consumption of processed and ultra-processed foods. According to the NOVA classification, UPFs are formulations made mostly from ingredients—some exclusively industrial—and additives, obtained through multiple industrial processes, and designed to be profitable, convenient, and hyper-palatable, and that tend to displace unprocessed or minimally processed foods in diet (10).

In Latin America, UPFs may account for up to 40% of children’s daily energy intake (11, 12). These foods often have high caloric density, added sugars, and excessive levels of sodium, total fats, and saturated fats (9, 13–15), which is why they have been associated with higher levels of adiposity, weight gain, increased blood pressure, and a less favorable lipid profile (9, 16–19). The mechanisms underlying these effects are multifactorial, involving lower satiety, higher glycemic responses, gut microbiota alterations, and exposure to chemical compounds formed during processing or released from packaging materials, which have been linked to endocrine disruption and increased risk for NCDs (9, 16, 20).

In Peru, over 60% of adults suffer from excess weight, as well as 10.8% of children under 5 years old (21), with this prevalence having doubled compared to what was observed in 2007 (22). Similarly, Peru experienced the highest per capita growth in the sale of ultra-processed foods in Latin America between 2009 and 2014 (23). In response to this issue, the Peruvian government enacted the Law to Promote Healthy Eating for Children and Adolescents (Law No. 30021), which includes a front-of-package nutrition labeling regulation that states that packaged foods and beverages that exceed specific thresholds of critical nutrients such as sugar, saturated fats, or sodium, or containing trans fats, must carry black octagonal warnings on the packaging and all forms of advertising. The thresholds for these critical nutrients were implemented gradually in two phases, with the first phase coming into effect in June 2019 and the second in September 2021.

Following the implementation of the first phase of Law No. 30021, a study identified that 31% of beverages and 62% of foods should carry an octagonal warning (24). However, that study excluded products specifically formulated for early childhood, such as breastmilk substitutes. Therefore, we conducted a study to assess and compare the nutritional profile of critical nutrients (sugars, saturated fats, sodium, and trans fats) in foods intended for children aged 0–3 years marketed in Metropolitan Lima, using national criteria and the PAHO model, as well as the presence and type of added sugars and non-caloric sweeteners. This study provides evidence that can inform policies, regulatory measures, and health promotion strategies aimed at improving the nutritional quality of foods consumed by infants and young children, a highly vulnerable group in which nutrition plays a critical role in health and long-term development.

## 2 Materials and methods

### 2.1 Study design

This was a cross-sectional, descriptive, and observational study. The study population included all breastmilk substitutes and foods intended for children aged 0–3 years marketed in Metropolitan Lima.

### 2.2 Selection of establishments

The 43 districts that comprise Metropolitan Lima were grouped into sectors based on the jurisdiction of the Integrated Health Networks of the Ministry of Health of Peru: Lima North, Lima Center, Lima East, and Lima South. Within each sector, supermarkets and hypermarkets from the eight chains with the largest market share in Peru (25) were identified, along with the hospital with the highest number of births in 2022, according to the National Unique Health Information Registry (REUNIS, in Spanish). In each sector, the following were selected: (a) one supermarket and one hypermarket (using convenience sampling); (b) all pharmacies around the hospital with the highest number of births; and (c) the online stores of the establishments listed in points (a) and (b).

### 2.3 Selection of products

The study included foods and beverages labeled for children under 36 months that did not require a medical prescription. For products available in different flavors, one flavor was randomly selected. The exclusion criteria comprised products without nutritional information and/or ingredient list; with damaged packaging that prevented proper reading of the labeling and/or nutritional composition; intended for infants who are premature, have low birth weight, or have specific medical conditions or disorders; and with inconsistent nutritional information (≥20% difference between the declared total energy and the energy calculated from the caloric content of carbohydrates, total fat, and proteins using the Atwater system).

### 2.4 Data collection

A snowball sampling technique was used for product collection until saturation was achieved. The first selected establishment was visited, and all products meeting the eligibility criteria were obtained. Subsequently, the second selected establishment was visited, and the remaining available products were acquired. This process was repeated at other establishments until all study products were collected.

Sample collection began with visits to all selected super- and hypermarkets, following this sequence: Lima Center, Lima North, Lima East, and Lima South. The process continued with the acquisition of products from the online stores of the selected establishments and concluded with the purchase of products from pharmacies near the selected hospitals in each sector. Once the products were acquired, they were photographed and stored according to the manufacturers’ instructions.

### 2.5 Data entry and processing

Nutritional composition and ingredient information from the labels of the products included in the study were entered into a spreadsheet designed in Microsoft Excel^®^ by one researcher (JCH). To ensure integrity and consistency, the entered information was verified by a second researcher (AA) using a 15% random sample of the products, selected with the *randomselect* command in Stata/SE version 17.0 (StataCorp, College Station, TX, United States) from their unique identification codes.

### 2.6 Outcomes

The following outcomes were analyzed (operational definitions for each outcome are detailed in Supplementary Table S1):

- the content of energy, proteins, total fats, carbohydrates, sugar, saturated fats, trans fats, and sodium per 100 g or 100 mL of product;
- the percentage of products with high levels of sugar, saturated fats, and sodium according to the thresholds established by Law No. 30021 (26) and those proposed by the Pan American Health Organization (PAHO) in the context of the update to Law No. 30021 (27) (Table 1).
- the percentage of products containing trans fats (greater than 0 g per 100 g or milliliters of product, and greater than 2 g per 100 g or milliliters of fat content);
- the percentage of products containing added sugars, as well as the type of added sugars used
- the percentage of products containing non-caloric sweeteners, as well as the type of non-caloric sweetener used.

**Table 1:** Technical parameters for classifying foods as “high” in sugar, saturated fats, and sodium according to Law No. 30021 and PAHO.

Classification	Law No. 30021*	PAHO^†^
High in sugar^‡^	≥10 g/100 g in solid foods or ≥5 g/100 mL in beverages	>0 kcal/100 kcal
High in saturated fats	≥ 4 g/100 g in solid foods or ≥ 3 g/100 mL in beverages	≥10 kcal/100 kcal
High in sodium	≥400 mg/100 g in solid foods or ≥100 mg/100 mL in beverages	≥60 mg/100 kcal

*Parameters according to the regulations of Peruvian Law No. 30021, Law to Promote Healthy Eating for Children and Adolescents (26). †Parameters proposed by the Pan American Health Organization (PAHO) within the context of the update to Law No. 30021 (27). ‡The parameters of Law No. 30021 consider total sugars, while the parameters proposed by PAHO consider added free sugars.

### 2.7 Statistical analysis

Qualitative variables were summarized using absolute (count) and relative (percentage) frequency measures. Quantitative variables were described as medians with interquartile ranges. Study results were presented as totals and stratified by product type (breastmilk substitutes, infant cereals, and pureed foods). Breastmilk substitutes were further analyzed according to the intended age group (<6 months, 6–12 months, and >12–23 months). Analyses were conducted using Stata/SE version 17.0 (StataCorp, College Station, TX, United States).

### 2.8 Ethics statement

The study protocol was submitted to the Institutional Review Board of the Asociación Benéfica PRISMA and was exempted from review as it involved an analysis of information declared on product labels and did not pose any risk to human subjects (Letter #CE0426.23).

## 3 Results

A total of 64 products collected between September and October 2023 were included in the analysis. These consisted of 39 breastmilk substitutes (60.9%), 13 infant cereals (20.3%), and 12 pureed foods (18.8%). Among the breastmilk substitutes, 12 were intended for infants under 6 months, 11 for infants 6–12 months, and 16 for children over 12–35 months.

### 3.1 Content of energy and macronutrients

Per 100 grams or milliliters, the analyzed products contained a median of 435.4 kcal (IQR: 295.3–489), 56.1 g of carbohydrates (IQR: 47.1–59.6), 10.4 g of protein (IQR: 4.5–15.0), and 15.1 g of total fat (IQR: 1.7–22.8). Breastmilk substitutes, regardless of the intended age group, were the products with the highest content of energy, protein, and total fat, while infant cereals had the highest carbohydrate content. Similarly, pureed foods were the products with the lowest median values for energy and macronutrients (Table 2).

**Table 2:** Content * of energy and macronutrients per 100 g or 100 mL.

Type of food		N	Energy (kcal)	Carbohydrates (g)	Proteins (g)	Total fats (g)
Breastmilk substitutes	Overall	39	486 (456–500)	56.2 (54.2–58.7)	14.3 (10.6–15.5)	22.0 (18.1–25.6)
	<6 mo^†^	12	513.5 (498.5–518)	56.5 (55.2–58.5)	10.4 (9.7–10.7)	26.6 (25.3–27.9)
	6–12 mo^†^	11	486 (461–490)	57.0 (54.0–58.4)	15.2 (14.3–15.5)	21.2 (19.5–22.1)
	>12–35 mo^†^	16	456.5 (391.5–483)	55.5 (52.8–59.3)	15.2 (13.3–16.1)	17.6 (9.7–21.9)
Infant cereals		13	393 (378–404.8)	80.5 (73.3–84.0)	9.0 (7.3–10.0)	2.5 (1.9–3.6)
Pureed foods		12	57.4 (52.5–65)	15.6 (13.9–17.2)	0.1 (0.0–0.6)	0.0 (0–0.1)
Total		64	435.4 (295.3–489)	56.1 (47.1–59.6)	10.4 (4.5–15.0)	15.1 (1.7–22.8)

* Medians and interquartile ranges (IQR) (25–75 percentiles). †Minimum suggested age according to the label.

### 3.2 Profile of critical nutrients

According to the parameters of Law No. 30021, the proportion of products rated as high in total sugars, sodium, and saturated fats was 74.5, 1.6, and 37.3%, respectively. When using the parameters proposed by PAHO, these percentages increased to 83% (added sugars), 6.3, and 41.3%, respectively. For both parameters, breastmilk substitutes were the most frequently classified as “high in sugar” and “high in saturated fats.” It should be noted that 40% of the breastmilk substitutes included in the study did not report information on sugar and/or saturated fat content on their labeling, and therefore were not considered in the analysis for these two critical nutrients. Finally, infant cereals were the food group most frequently classified as “high in sodium” using the PAHO parameters (15.4%) (Table 3).

**Table 3:** Content* and profile of critical nutrients.^**†**^

Type of food	Sugar			Sodium			Saturated fats			Trans fats ^§§^		
	Content^‡^ (g/100 g)	“High in”^§^		Content (mg/100 g)	“High in”		Content (g/100 g)	“High in”		Content (g/100 g)	>0 g/100 g	>2 g/100 g of fat
		Law No. 30021	PAHO		Law No. 30021	PAHO		Law No. 30021	PAHO			
		n/N (%)	n/N (%)		n/N (%)	n/N (%)		n/N (%)	n/N (%)		n/N (%)	n/N (%)
Breastmilk substitutes	52.2 (31.0–57.0)	21/22 (95.5%)	15/22 (68.2%)	200 (170–240)	1/39 (2.6%)	2/39 (5.1%)	7.9 (3.7–8.8)	18/26 (69.2%)	19/24 (79.2%)	0.0 (0.0–0.2)	7/20 (35.0%)	1/20 (5.0%)
<6 mo†	56.1 (54–58.6)	6/6 (100.0%)	3/6 (50.0%)	170 (159.5–182.5)	0/12 (0.0%)	0/12 (0.0%)	9.2 (8–10.9)	5/6 (83.3%)	5/6 (83.3%)	0.1 (0.0–0.18)	3/5 (60.0%)	0/5 (0.0%)
6–12 mo†	53.7 (51.5–57)	6/6 (100.0%)	4/6 (66.7%)	220 (190–240)	0/11 (0.0%)	0/11 (0.0%)	7.7 (2.5–9)	4/6 (66.7%)	5/6 (83.3%)	0.00 (0.0–0.14)	2/5 (40.0%)	0/5 (0.0%)
>12–35 mo†	33.4 (27–37.3)	9/10 (90.0%)	8/10 (80.0%)	232.5 (177.8–253)	1/16 (6.3%)	2/16 (12.5%)	7.3 (3.7–8)	9/14 (64.3%)	9/12 (75.0%)	0.00 (0.0–0.0)	2/10 (20.0%)	1/10 (10.0%)
Infant Cereals	17 (9.1–21.0)	8/13 (61.5%)	12/13 (92.3%)	45 (17–150)	0/13 (0.0%)	2/13 (15.4%)	0.6 (0.4–0.8)	1/13 (7.7%)	0/13 (0.0%)	0.00 (0.0–0.0)	1/13 (7.7%)	0/13 (0.0%)
Pureed Foods	10.8 (9.3–12.8)	6/12 (50.0%)	12/12 (100.0%)	2.7 (1.5–9.1)	0/12 (0.0%)	0/12 (0.0%)	0.0 (0.0–0.0)	0/12 (0.0%)	0/9 (0.0%)	0.00 (0.0–0.0)	0/4 (0.0%)	0/4 (0.0%)
Total	20.0 (9.5–51.5)	35/47 (74.5%)	39/47 (83.0%)	170 (23.5–223.5)	1/64 (1.6%)	4/64 (6.3%)	2.5 (0.6–8.0)	19/51 (37.3%)	19/46 (41.3%)	0.00 (0.0–0.0)	8/37 (21.6%)	1/37 (2.7%)

*Medians and interquartile ranges (IQR) (25–75 percentiles). †“n/N” represents the number of items that are above the threshold established (n) out of the total number of items assessed (N). ‡Total sugars. § The parameters of the Law No. 30021 consider total sugars, while the parameters proposed by PAHO consider added free sugars. §§According to current regulations in Peru, foods must not contain trans fats from partial hydrogenation. However, a limit of 2 g/100 g of fat is allowed when “the trans fat content has been reduced as much as possible according to the technology used for processing, and there is no technological substitution for its complete removal” §§§Minimum suggested age according to the label.

When the PAHO parameters were used to identify products excessive in sugars, sodium or saturated fats, the proportion of products with no warning labels dropped to less than half compared to the current parameters of the Law No. 30021 (Table 4).

**Table 4:** Proportion of products requiring warning labels by PAHO and Law No. 30021 (phase 2).

Number of warnings	Law No. 30021 (*N* = 45)	PAHO (*N* = 42)	Prevalence ratio (PAHO vs Law No. 30021)
0	26.7%	9.5%	0.36
1	37.8%	50%	1.32
2	35.6%	40.5%	1.14
3	0.0%	0.0%	–
One or more warnings	73.3%	90.5%	1.23

### 3.3 Presence of trans fats

A total of 19 breastmilk substitutes (48.7%) and 8 pureed foods (66.7%) did not declare information on trans fat content on their labels. Among the remaining 37 products, 8 (21.6%) had a trans fats content greater than 0 g, and one of them exceeded 2 g of trans fats per 100 g/mL of fat content. The presence of trans fats was observed in seven breastmilk substitutes and one infant cereal (Table 3).

### 3.4 Presence and type of added sugars

100% of breastmilk substitutes, 53.9% of infant cereals, and 66.7% of pureed foods reported one or more added sugars in their ingredient lists. Among breastmilk substitutes, the most frequently added sugars were lactose (69.2%) and maltodextrin (41%). Lactose was particularly common in products intended for infants under 6 months (91.7%), while maltodextrin predominated in products aimed at the >12–35 months age group. Sugar or sucrose was the most commonly added sugar in infant cereals (30.8%) and pureed foods (66.7%), and it was present in 18.8% of breastmilk substitutes intended for the >12–35 months age group (Table 5).

**Table 5:** Presence and type of added sugars [n/N (%)].*

Added sugar	Total	Breastmilk substitutes				Infant cereals	Pureed foods
		Overall	<6 mo^†^	6–12 mo^†^	>12–35 mo^†^		
Lactose	28/64 (43.8%)	27/39 (69.2%)	11/12 (91.7%)	8/11 (72.7%)	8/16 (50.0%)	1/13 (7.7%)	0/12 (0.0%)
Maltodextrin	17/64 (26.6%)	16/39 (41.0%)	4/12 (33.3%)	4/11 (36.4%)	8/16 (50.0%)	1/13 (7.7%)	0/12 (0.0%)
Sugar/sucrose	15/64 (23.4%)	3/39 (7.7%)	0/12 (0.0%)	0/11 (0.0%)	3/16 (18.8%)	4/13 (30.8%)	8/12 (66.7%)
Corn syrup	8/64 (12.5%)	8/39 (20.5%)	3/12 (25.0%)	2/11 (18.2%)	3/16 (18.8%)	0/13 (0.0%)	0/12 (0.0%)
Glucose syrup	5/64 (7.8%)	5/39 (12.8%)	1/12 (8.3%)	2/11 (18.2%)	2/16 (12.5%)	0/13 (0.0%)	0/12 (0.0%)
Honey	2/64 (3.1%)	0/39 (0.0%)	0/12 (0.0%)	0/11 (0.0%)	0/16 (0.0%)	2/13 (15.4%)	0/12 (0.0%)
Fruit concentrate	1/64 (1.6%)	0/39 (0.0%)	0/12 (0.0%)	0/11 (0.0%)	0/16 (0.0%)	0/13 (0.0%)	1/12 (8.3%)
Maltose	1/64 (1.6%)	0/39 (0.0%)	0/12 (0.0%)	0/11 (0.0%)	0/16 (0.0%)	1/13 (7.7%)	0/12 (0.0%)
Panela (unrefined cane sugar)	1/64 (1.6%)	0/39 (0.0%)	0/12 (0.0%)	0/11 (0.0%)	0/16 (0.0%)	1/13 (7.7%)	0/12 (0.0%)
One or more added sugars	54/64 (84.4%)	39/39 (100%)	12/12 (100%)	11/11 (100%)	16/16 (100%)	7/13 (53.9%)	8/12 (66.7%)
No added sugars	10/64 (15.6%)	0/39 (0%)	0/12 (0.0%)	0/11 (0.0%)	0/16 (0.0%)	6/13 (46.2%)	4/12 (33.3%)

* “n/N” represents the number of items that meet the criteria (n) out of the total number of items assessed (N). †Minimum suggested age according to the label.

### 3.5 Presence and type of non-caloric sweeteners

None of the infant cereals or pureed foods declared the presence of non-caloric sweeteners in their composition. Among the breastmilk substitutes, two products (3.1%) intended for children >12–35 months declared containing non-caloric sweeteners, specifically steviol glycosides (E960A) and sodium saccharin (E954).

## 4 Discussion

Our study aimed to assess and compare the nutritional profile of critical nutrients (sugar, saturated fats, sodium, and trans fats) in foods intended for children aged 0–3 years marketed in Metropolitan Lima, using the criteria established by Law No. 30021—which regulates front-of-package warning labels in Peru—and the nutrient profile model developed by PAHO. The purpose of this study was to provide key evidence to inform regulatory actions and health promotion strategies aimed at protecting the diets of infants and young children, a highly vulnerable group.

Overall, we observed a high proportion of foods with excessive content of sugar and saturated fats. When using the parameters established by Law No. 30021, 74.5% of products were classified as “high in sugars (totals)” and 37.3% as “high in saturated fats.” These percentages increased to 83% for “high in sugars (added)” and 41.3% for “high in saturated fats” when applying the PAHO parameters.

Studies in Latin America (14, 28–31) have reported varying percentages of infant foods with excessive content of critical nutrients. Regarding excessive sugar content, percentages have ranged from 37% in Brazil (14), to 91% in Uruguay (28), while the percentage of infant foods with excessive saturated fat content has varied from 11% in Chile (14) to 50% in Uruguay (30). This variability could be attributed to differences in regulation implementation periods across countries, the use of different definitions of infant foods, eligibility criteria, and sources of information, as well as by the application of various technical parameters to classify foods with excessive critical nutrient content.

Regarding this matter, Borges et al. (31) analyzed a sample of infant foods using six different technical parameters, demonstrating that the percentage of foods classified as “high in sodium” and “high in saturated fats” could be up to two or three times higher, and that the percentage of foods “high in sugar” could be up to 35% higher depending on the technical parameter used. Similarly, Karageuzián et al. (28) observed that the percentage of infant foods containing at least one critical nutrient increased from 74% using the parameters established by Uruguay’s national regulations to 96% when the parameters proposed by PAHO were used. Consistent with our study, both authors (28, 31) reported that using the technical parameters proposed by PAHO allowed for the identification of a greater number of foods with excessive levels of critical nutrients.

The choice of a specific technical parameter or nutrient profile model (NPM) is crucial for food policies, as it can affect the proportion of foods that should carry warning nutritional labeling and could be subject to advertising and marketing restrictions (31, 32). Consequently, a less stringent NPM might misinform consumers, encourage the consumption of less healthy foods, and impact food policies and the achievement of nutritional goals (31, 32). At the same time, the use of different NPMs among countries can lead to inconsistencies, confusion, and hinder comparability and tracking of progress (32, 33).

In this context, PAHO established evidence-based regional criteria in 2016, developed with the involvement of nutrition experts (34). This nutrient profile was designed based on the population intake targets proposed by WHO and FAO to prevent obesity and non-communicable chronic diseases, goals that were defined after a review of evidence on critical nutrients affecting public health (34).

However, most Latin American countries have developed their own models or modified the original criteria proposed by PAHO with input from various sectors, including the food industry (32, 35). On this matter, a recent systematic review (36) indicates that NPMs developed with participation from the food industry often have less stringent criteria compared to those involving independent academic experts.

In the case of Peru, a study documented multiple changes in the technical parameters during the formulation of the Healthy Eating Law and its regulations. These changes were related, among other factors, to the lack of political consensus and the active opposition of the food industry, which exerted pressure to delay implementation and to make the regulation more flexible. As a result, a more flexible approach was ultimately adopted, based on the technical parameters established in Chile (37).

By contrast, the thresholds defined by PAHO are stricter, as they set lower limits on the content of sugars, saturated fats, and sodium compared to other national regulations (31). This greater rigor aims to provide a higher level of health protection, especially during childhood, and to serve as a technical reference standard for designing policies that promote healthier food environments (34).

The use of warning nutritional labeling, as part of a comprehensive policy that includes restrictions on the advertising and marketing of foods high in critical nutrients, has shown a positive impact on purchasing decisions and the reformulation of the nutritional content of processed foods in Latin American countries (35). For example, 2 years after the implementation of the first phase of the national warning nutritional labeling policy in Peru, a study reported a decrease in sugar content in beverages, which in turn led to a reduction from 59 to 31% in the percentage of products that would require high-sugar labeling, compared to the phase before implementation. Similarly, a reduction in saturated fat content in foods was observed, decreasing the percentage of products high in saturated fats from 82 to 62% (24).

On the other hand, an important aspect in the context of implementing a warning nutrition labeling system concerns the criteria that determine which foods and beverages are subject to regulation (38). Often, infant formulas are considered “foods for special dietary uses (FSDU)” and are not included in the scope of warning nutrition labeling policies (38). However, these products were one of the main contributors to the high proportion of foods high in sugar and saturated fats observed in our study. Therefore, their exclusion could represent a limitation on consumers’ right to be adequately informed and discourage the food industry from reformulating their nutritional content (24).

Added sugars were present in all breastmilk substitutes, 67% of infant cereals, and 54% of pureed foods. Additionally, between 74.5 and 83% of the products included in our study had excessive sugar content. Excessive sugar content in infant foods has been widely reported by various studies around the world (15, 39–41), and is a cause for concern because these are calorically dense, non-nutritive elements, and repeated and excessive exposure can reinforce the innate preference for sweet taste in children, affect the overall diet quality, displace the consumption of more nutritionally valuable foods, promote excessive weight gain, and increase the risk of non-communicable diseases (42, 43). For this reason, various guidelines, such as the WHO complementary feeding guidelines and the U. S. Dietary Guidelines, now recommend that children under 2 years old avoid consuming foods high in sugar or with added sugars, and sweetened beverages (44, 45).

With respect to sodium content, less than 10% of the products included in our study were classified as high in sodium. These findings were consistent with those reported by Saavedra-Garcia et al. (24) in a study conducted in Peru to compare the impact of the first phase of the implementation of Law No. 30021 on the content of critical nutrients in foods and beverages. In that study, the researchers reported a low number of high-sodium products, even before the implementation of the Law. Interestingly, the authors suggest that this effect could be an indirect result of the prior implementation of regulations on sodium content in neighboring countries like Chile, which may have led to the reformulation of products also distributed by transnational companies in the Peruvian market (24).

Concerning saturated fat content, around 40% of the included products had excessive levels. However, there were significant differences within the groups. While excess saturated fat was present in less than 10% of cereals and in none of the pureed foods, between 70 and 80% of breastmilk substitutes had excessive levels of this critical nutrient. Additionally, the median saturated fat content in breastmilk substitutes was nearly 8 g (data not shown), which was even higher than the median observed for processed foods before the implementation of Law No. 30021 (24).

This situation is particularly concerning considering that, in Peru, the consumption of breastmilk substitutes among children under 3 years of age almost doubled, increasing from approximately 8% in 2010 to 14% in 2019 (46, 47). The increase was especially pronounced among infants under 6 months of age, where prevalence rose from 19.3 to 52.9% over the same period. Such trends may contribute to early and excessive intake of saturated fats, contradicting the WHO recommendation to reduce their consumption in children to less than 10% of total energy intake (48). The significant contribution of breastmilk substitutes to saturated fat intake has also been documented in countries such as the United States, where 90% of the saturated fats consumed by children under 1 year of age came from these types of products (49).

The growing consumption of ultra-processed foods observed in Peru (23) and the sustained increase in childhood overweight, which has doubled over the past 15 years to affect 10.8% of children under 5 years of age (21, 22), may reflect a food environment that promotes unhealthy dietary patterns from the earliest stages of life, contributing to the persistence of excess weight with significant health and economic consequences. In this regard, it is estimated that between 2025 and 2092, the total direct and indirect costs attributable to childhood and adolescent overweight and obesity in Peru will amount to 210.6 billion dollars (50), underscoring the urgency of implementing policies and strategies aimed at improving the nutritional quality of foods intended for this population.

Nonetheless, we must consider some potential limitations of our study. First, products were acquired from supermarkets and selected pharmacies. This may have limited the identification of products distributed through other channels, such as those marketed as part of social assistance programs, sold in bulk in low-income communities, or commercialized via e-commerce platforms. However, we believe that the stores selected in our study represent the locations with the highest sales volume of these products and thus reflect the infant foods with the greatest availability for the Peruvian population. Future studies could expand this scope by incorporating multi-channel sampling strategies and laboratory analyses to comprehensively assess the nutritional quality of breastmilk substitutes and foods across all socioeconomic contexts.

Secondly, our analysis was based on information declared on the product labels. In this regard, we found that 40% of breastmilk substitutes did not report sugar and/or saturated fat content, making it impossible to evaluate the nutritional profile for these two critical nutrients in those products. Additionally, some studies (51, 52) have reported discrepancies between the nutritional information declared on infant food labels and the analytical values derived from laboratory tests. Therefore, there is a need to update nutritional labeling regulations in Peru to mandatorily include reporting of critical nutrients such as sugar, trans fats, and saturated fats, and, at the same time, future studies are needed to confirm our findings through laboratory determinations.

Finally, we only selected one unit of each product, regardless of size or flavor. This could be a limitation considering that some labeling characteristics might change according to the packaging dimensions. Similarly, different flavors of the same product could vary in their nutritional composition, although we believe that this would not significantly alter the critical nutrients of interest for our study.

## 5 Conclusion

Our study identified that most foods marketed for children under 3 years old in Lima Metropolitan Area contain added sugars and excessive amounts of critical nutrients such as sugars (totals or added) and saturated fats. Breastmilk substitutes were among the main products that contributed to these results. Finally, the use of the technical parameters proposed by PAHO allowed for the identification of a higher number of products high in critical nutrients compared to the current parameters established by Law No. 30021.

## Data Availability

The datasets presented in this study can be found in online repositories. The names of the repository/repositories and accession number(s) can be found at: https://doi.org/10.6084/m9.figshare.29914136.v1.
