# Supplementary material for: Comparison between national and PAHO criteria for assessing the nutritional profile of foods intended for children aged 0–3 years marketed in Lima, Peru
Source: Front Nutr. 2025 Sep 9;12:1592172. doi: 10.3389/fnut.2025.1592172 (PMC12454305; doi:10.3389/fnut.2025.1592172)
Supplement: Supplementary file 1 [file Table_1.docx]

Comparison Between National and PAHO Criteria for Assessing the Nutritional Profile of Foods Intended for Children Aged 0–3 Years Marketed in Lima, Peru.

Supplementary Material

**Supplementary Table S1.** Operational definitions of the study outcomes.

| **Outcome** | **Operational definition** |
| --- | --- |
| **Content of energy and macronutrients per 100 g/mL** | Values obtained from the information declared on the product label. Energy values were expressed in kilocalories, while protein, fat, and carbohydrate values were expressed in grams. Values reported using different reference weights (e.g., per serving) were converted to 100 g/ml of product by multiplying the reported value by 100 and dividing by the reference weight. Data expressed through medians and interquartile ranges (IQR). |
| **Content of critical nutrients**  **(sodium, sugar, saturated fat, and trans fats) per 100 g/mL** | Values obtained from the information declared on the product label. Sodium values were expressed in milligrams, while sugar, saturated fat, and trans fats values were expressed in grams. Values reported using different reference weights (e.g., per serving) were converted to 100 g/ml of product by multiplying the reported value by 100 and dividing by the reference weight. Data expressed through medians and interquartile ranges (IQR). |
| **Profile of critical nutrients (sodium, sugar and saturated fat)** | Products were classified as 'High in' sugar, saturated fat, and sodium according to the technical parameters established by Law No. 30021 and those proposed by the Pan American Health Organization (PAHO) (see Table 1 in the main manuscript). For products without information on saturated fat content, they were considered 'low in saturated fat' according to Law No. 30021 when total fat values were reported as <4 g/100 g in solid foods and <3 g/100 g in beverages, and 'low in saturated fat' according to PAHO technical parameters when total fat values were reported as zero. |
| **Presence of trans fats** | The information was obtained from the product label. The presence of trans fats was evaluated using two cut-off points: > 0 g per 100 g/ml of product (Law No. 30021 states that foods with trans fats must carry a nutritional warning octagon), and > 2 g per 100 g/ml of fat (Peruvian regulations exceptionally allow this limit when there is no technological substitute for their complete elimination). |
| **Presence and type of added sugars** | The information was obtained from the ingredient list on the product. The presence of various types of added sugar was identified, including maltodextrin, sugar, glucose, fructose, lactose, galactose, sucrose, maltose, trehalose, caramel, syrups, honey, and/or fruit concentrate |
| **Presence and type of non-caloric sweeteners** | The information was obtained from the product label or ingredient list. Non-caloric sweeteners were considered to include the presence of aspartame, sucralose, saccharin, neotame, potassium acesulfame, cyclamates, alitame, advantame, stevia, Luo Han Guo (monk fruit), thaumatin, pentadin, monellin, and brazzein. |
